# The Potential of Phage Therapy against the Emerging Opportunistic Pathogen *Stenotrophomonas maltophilia*

**DOI:** 10.3390/v13061057

**Published:** 2021-06-03

**Authors:** Jaclyn G. McCutcheon, Jonathan J. Dennis

**Affiliations:** Department of Biological Sciences, University of Alberta, Edmonton, AB T6G 2E9, Canada; jgmccutc@ualberta.ca

**Keywords:** *Stenotrophomonas maltophilia*, bacteriophage, phage therapy, antibiotic resistance

## Abstract

The isolation and characterization of bacteriophages for the treatment of infections caused by the multidrug resistant pathogen *Stenotrophomonas maltophilia* is imperative as nosocomial and community-acquired infections are rapidly increasing in prevalence. This increase is largely due to the numerous virulence factors and antimicrobial resistance genes encoded by this bacterium. Research on *S. maltophilia* phages to date has focused on the isolation and in vitro characterization of novel phages, often including genomic characterization, from the environment or by induction from bacterial strains. This review summarizes the clinical significance, virulence factors, and antimicrobial resistance mechanisms of *S. maltophilia*, as well as all phages isolated and characterized to date and strategies for their use. We further address the limited in vivo phage therapy studies conducted against this bacterium and discuss the future research needed to spearhead phages as an alternative treatment option against multidrug resistant *S. maltophilia*.

## 1. Introduction

The increasing prevalence of broad-spectrum antimicrobial resistance in bacterial infections worldwide is a global health concern. Use and misuse of antimicrobials have driven the evolution of resistant bacteria and the effectiveness of current antibiotics against bacterial pathogens is rapidly declining, created the risk of a post-antibiotic era in the near future; reports estimate that antimicrobial resistant bacterial infections will cause 10 million deaths annually worldwide by the year 2050 with significant socio-economic impacts if alternative treatment options are not discovered [1,2].

*Stenotrophomonas maltophilia* is one bacterium of concern that is emerging as a multidrug resistant opportunistic nosocomial pathogen. *S. maltophilia* infections are difficult to treat with conventional antibiotics due to numerous chromosomally encoded antimicrobial resistance mechanisms [3]. The use of bacterial viruses, or bacteriophages, as an alternative treatment is an attractive option due to the specificity of these viruses to their host. In this review, we will first briefly summarize the currently existing research on *S. maltophilia* pathogenicity mechanisms and then examine the potential of phage therapy as an alternative treatment option to antibiotics in light of the extreme antibiotic resistance of this bacterial pathogen.

## 2. *Stenotrophomonas maltophilia*

**Figure 1:**
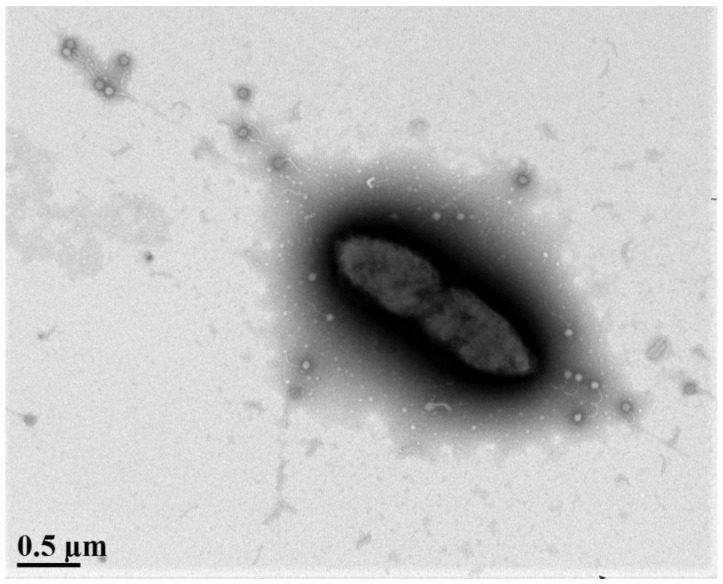
Transmission electron micrograph of *S. maltophilia* cell attacked by phages. *S. maltophilia* strain D1585 with numerous DLP1 bacteriophage [11] virions binding to type IV pili that are protruding from the cell. Cells and phages were stained with 2% phosphotungstic acid and visualized at 18,000-fold magnification by transmission electron microscopy (McCutcheon, J. G. and Oatway, A.; University of Alberta).

*S. maltophilia* is a Gram-negative obligate aerobe that is motile due to the presence of polar flagella, as well as type IV pili that aid in twitching motility and biofilm formation [3,4,5] (Figure 1). This bacteria is ubiquitous in the environment, often having beneficial interactions with plants, both on their surface and in the rhizosphere [6]. First isolated as *Bacterium bookeri* in 1943 by J. L. Edwards, this species was originally named *Pseudomonas maltophilia* by Hugh and Ryschenko in 1961 [7], followed by controversial reclassification into the genus *Xanthomonas* in 1983 [8] before finally being given its own genus in 1993 [9]. *S. maltophilia* is now one of 20 species in the genus *Stenotrophomonas* currently listed in the NCBI taxonomy browser. Strains with 16S rRNA gene sequence similarities greater than 99.0% have been grouped into the ‘*S. maltophilia* complex’ (Smc) to encompass the genetic heterogeneity and diversity of these bacteria [10].

The genus name *Stenotrophomonas*, translating as “narrow one who feeds” was meant to reflect the perceived limited nutritional spectrum of these bacteria, however further research has demonstrated the vast metabolic diversity and intraspecific heterogeneity within this genus [6,12]. We now know that *S. maltophilia* bacteria are capable of utilizing a wide range of carbon sources, have an intrinsic resistance to heavy metals, and tolerate nutrient-poor environments, allowing them to survive and persist in many undesirable conditions [3,6,13]. In addition to the ability to metabolize a variety of organic compounds, such as phenolics and xenobiotics, *Stenotrophomonas* species are not phytopathogenic, unlike the closely related genera *Xanthomonas* and *Xylella*, and can promote plant productivity via the expression of the plant growth hormone indole-3-acetic acid (IAA) [6]. These properties make *S. maltophilia* a desirable candidate for the bioremediation of soil contaminated with heavy metals or pesticides and for biotechnical applications in agriculture to promote plant productivity [6,13,14,15], however, the ability of *S. maltophilia* to cause disease in humans discourages their use in agriculture [6,13].

### 2.1. Clinical Prevalence and Significance

*S. maltophilia* is the most prominent species within this genus and is of rising concern due to its ability to cause human disease [3,6]. The significant genetic and phenotypic heterogeneity within *S. maltophilia* populations allows these bacteria to adapt rapidly under changing selective pressures in both a clinical and environmental setting [3,5,10,13,16]. This high genetic diversity can be observed even between isolates from the same hospital [17], with higher mutation frequencies observed in clinical isolates compared to those from environmental sources [18]. Global surveillance programs began tracking the prevalence and clinical significance of *S. maltophilia* in the late 1990s; the frequency of *S. maltophilia* occurrence among bacterial isolates from all sources ranged from 0.8% to 1.4% during the 1997 to 2003 time period and increased to prevalence rates of 1.3% to 1.68% in the years 2007 to 2013 [19]. Current data from the Canadian Ward Surveillance Study (CANWARD) identified *S. maltophilia* at a frequency of 2.98% in the nearly 3000 pathogens isolated from hospitalized patients in the year 2018 [20]. These data suggest an increasing trend in *S. maltophilia* infections in recent years.

Recently, a comprehensive genome-based phylogenetic analysis of an international collection of 1305 Smc isolates from 22 countries, 87% of which were from clinical origin, was undertaken to understand the global population structure of the Smc, identify human-associated lineages and the potential for global and local spread [21]. The genome collection clustered into 23 monophyletic lineages named Sgn1-Sgn4 and Sm1-Sm18, with lineage Sgn4 most distantly related to the rest of the strains. The largest lineage was Sm6 and comprises the highest rate of human-associated strains. Key virulence and antibiotic resistance genes, including multiple efflux pumps, were found in all lineages, however, some genes were unequally distributed. Notably, through genetic diversity analysis, the authors identified hospital-linked clusters of strains collected within short time intervals, suggesting potential direct or indirect human-to-human transmission. Although average nucleotide identity between the 23 lineages clearly distinguishes them, the authors note that it is also below the threshold considered to define a species, suggesting further studies to revise the taxonomic assignments and nomenclature for this group are required [21].

Numerous virulence factors including biofilm formation and the secretion of hydrolytic enzymes that allow environmental *S. maltophilia* isolates to colonize plant surfaces and compete with other soil microbes are also important for the colonization of medical devices and patients [6]. Listed by the World Health Organization as one of the leading drug-resistant nosocomial pathogens worldwide [22], this opportunistic pathogen is rapidly increasing in prevalence in nosocomial and community-acquired infections worldwide, passing easily between immunocompromised patients and health care providers through direct contact and cough-generated aerosols [3]. Most commonly associated with respiratory infections, *S. maltophilia* can also cause severe bacteremia, meningitis, endocarditis, pneumonia, osteomyelitis, endophthalmitis, and catheter-related bacteremia/septicemia [3]. Numerous risk factors for *S. maltophilia* infection include chronic respiratory disease, the presence of indwelling devices, underlying malignancy, a compromised immune system, prior use of antibiotics, and prolonged hospital or ICU stay [3,23]. *S. maltophilia* can adhere to and form biofilms on plastic surfaces, allowing colonization of many humid hospital surfaces, as well as intravenous cannulae, prosthetic devices, and nebulizers [3,19]. In response to starvation or stress, these bacteria are also able to form ultramicrocells that can pass through 0.2 µm filters similar to point-of-use water filtration used in hospital showers, potentially becoming a source for hospital-acquired infection [24]. In addition, tolerance to antiseptics and hydrogen peroxide-based disinfectants is provided by the presence of *qacEΔ1* and *katA* genes in many isolates [3,21,25,26], making *S. maltophilia* well-equipped to persist and spread in hospital settings.

Patients with cystic fibrosis are at greater risk for *S. maltophilia* infections than the general population with prevalence increasing significantly in recent decades [27,28]; data collected in 2019 by Cystic Fibrosis Canada and the Cystic Fibrosis Foundation shows that *S. maltophilia* were present in the airways of 14% and 11.9% of patients with cystic fibrosis in Canada and the USA, respectively [27,29]. Although the pathogenicity of *S. maltophilia* and its role as a colonizer of cystic fibrosis lungs or causative agent of disease has been unclear [3,30,31], retrospective studies indicate that this bacterium is a marker of lung disease severity [31,32,33]. *S. maltophilia* isolates are highly immunostimulatory and have been shown to significantly increase expression of the potent pro-inflammatory cytokine TNF-α in a murine lung, likely contributing to airway inflammation and the development of pneumonia [34]. Of particular concern is the interaction between *S. maltophilia* and other pathogens in polymicrobial infections of the cystic fibrosis lung, specifically *Pseudomonas aeruginosa,* one of the most prominent pathogens found in cystic fibrosis patients [31,33,35,36]. Studies show cooperativity between these bacterial species, with each bacterium benefitting from the presence of the other. Reports indicate that polymicrobial infection with *S. maltophilia* and *P. aeruginosa* in patients with cystic fibrosis may increase virulence, as patients with co-infections had significantly higher mortality rates than those with monoculture infections [37]. Early studies in vitro showed that *S. maltophilia* can encourage growth of *P. aeruginosa* in the presence of β-lactam antibiotics due to secretion of β-lactamases, indirectly contributing to disease progression [36]. Additionally, interspecies communication has been observed to occur through quorum sensing; *S. maltophilia*-produced diffusible signal factor (DSF) is recognized by *P. aeruginosa*, resulting in significantly altered biofilm structure and virulence factor expression, including increased tolerance to cationic antimicrobial peptides [38]. Although no *S. maltophilia* strain has been reported to produce an *N*-acyl homoserine lactone (AHL) quorum sensing signaling molecule, *S. maltophilia* is also capable of sensing *P. aeruginosa*-produced AHL using its LuxR solo SmoR (Smlt1839) protein, leading to changes in virulence factors such as swarming motility [39]. This synergy can also be observed in vivo; co-microbial infections with *P. aeruginosa* resulted in significantly higher *S. maltophilia* bacterial loads in the murine lung and this increase was directly correlated with live *P. aeruginosa* cell density [35].

### 2.2. S. maltophilia Virulence Factors

**Figure 2:**
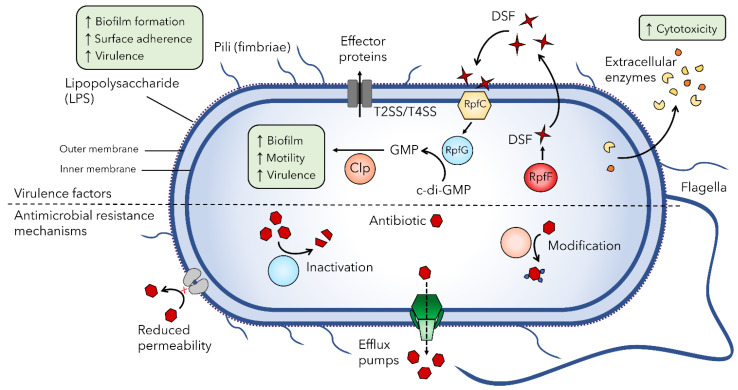
*S. maltophilia* pathogenicity and antibiotic resistance mechanisms. *S. maltophilia* encodes many virulence factors that contribute to its pathogenicity. Hydrolytic enzymes (yellow and orange shapes) released from the cell and secreted effector proteins contribute to cytotoxicity. Surface structures such as LPS, flagella, type IV pili, and SMF-1 fimbriae help the bacterium adhere to surfaces and form antibiotic resistant biofilm communities, contributing to increased virulence. Quorum sensing via diffusible signal factors (DSF, red stars) induces downstream gene expression shown to increase biofilm, motility and the virulence factors described. The extreme multidrug resistance of this bacterium is due to numerous mechanisms, including reduced membrane permeability, numerous chromosomally encoded efflux pumps, β-lactamases, and aminoglycoside-modifying enzymes. Antimicrobial molecules are represented by red hexagons.

Although *S. maltophilia* is not considered a highly virulent pathogen to healthy individuals, increasing nosocomial infection rates are of concern. Pathogenesis of infections caused by this bacterium involves numerous virulence factors and the ability to form biofilms on abiotic surfaces and host cells [3,40]. Production of these virulence factors has been linked to iron availability in the infection environment; under iron-restricted conditions or in a ferric uptake regulator *fur* mutant, *S. maltophilia* K279a produces more dense biofilms, increased amounts of exopolysaccharide and DSF, and is more virulent than in wildtype or iron-rich conditions [41]. This is concerning because in the lung iron is not biologically available due to lactoferrin sequestration, potentially contributing to increased pathogenicity of *S. maltophilia* infections [41,42]. Analyses of early whole genome sequencing data of *S. maltophilia* strain K279a identified numerous putative virulence and antimicrobial resistance genes by homology to known factors in other pathogens [43]. Research has since sought to characterize cell-associated and extracellular virulence factor mechanisms and their role in the pathogenesis of *S. maltophilia*. Specifically, research into the mechanisms of adherence to and colonization of medical devices and epithelial cells, which allows the formation of antibiotic and immune resistant biofilms that are characteristic of *S. maltophilia* infections and disease progression, is of utmost importance [3,40,44]. The main virulence factors and antibiotic resistance mechanisms in *S. maltophilia* discussed below are summarized in Figure 2.

*S. maltophilia* isolates express numerous cell-associated virulence factors on their surface. The outer lipopolysaccharide (LPS) layer of *S. maltophilia* is structurally diverse between strains [34,45] and plays an important role in colonization and virulence in a host; research has shown that *spgM* mutants deficient in the assembly of O-polysaccharide are unable to colonize rat lungs and are completely avirulent in this animal model, showing no histopathological changes [46]. Additionally, *spgM* mutants were susceptible to complement-mediated killing, exhibiting increased sensitivity to human serum compared to wildtype [46]. The *rmlBACD* and *xanAB* operons that are involved in the synthesis of lipopolysaccharide and exopolysaccharide also contribute to biofilm formation, with defective LPS production associated with decreased biofilm formation on hydrophobic surfaces [47].

Motility and fimbriae structures are also important for virulence and contribute to the formation of biofilms through adherence. The flagella is an important immunogenic structure that is found at the pole of the cell and is responsible for swimming motility [47,48]. Studies show that the *S. maltophilia* flagella plays a role in adherence to abiotic plastic surfaces [48] as well as mouse tracheal mucus [49], and flagella-deficient mutants have significantly reduced adherence to human bronchial epithelial cell monolayers obtained from cystic fibrosis patients [50]. The type 1 fimbriae SMF-1 is also implicated in adhesion to epithelial cells [51]. This adhesion, as well as adherence to abiotic surfaces, was inhibited by anti-SMF-1 antibodies. Also involved in haemagglutination and biofilm formation, SMF-1 fimbriae were identified in all clinical isolates tested [51], and were absent from *S. maltophilia* isolates of environmental origin [52], suggesting a role in respiratory tract infection in cystic fibrosis patients. Lastly, the type IV pilus is an important virulence factor on the bacterial cell surface that plays a role in motility, adherence to biotic and abiotic surfaces, and biofilm formation in many bacterial pathogens [53]. In *S. maltophilia*, type IV pili-mediated twitching motility has been correlated with increased biofilm mass in cystic fibrosis isolates [5,47] and although the majority of clinical isolates are twitching positive [5,54], the role of type IV pili in virulence has not been studied in depth in *S. maltophilia*.

Numerous secreted enzymes and proteins have been studied for their contribution to *S. maltophilia* pathogenesis as extracellular virulence factors. These include proteases, lipases, phospholipases, esterases, nucleases, haemolysins, cytotoxins, and siderophores [3,40,43]. The production of these enzymes provides a competitive advantage in unfavorable conditions, such as the rhizosphere, but also contributes to cytotoxicity [6,40,55]. The major protease StmPr1 associated with tissue destruction and evasion of host defense mechanisms [56], along with serine proteases StmPr2 and StmPr3, were recently shown to be substrates of the Xps type II secretion system (T2SS) in *S. maltophilia* [57,58,59]. These secreted proteases are largely responsible for Xps-mediated detrimental morphological and cytotoxic effects on lung epithelial cells, demonstrating the significance of the Xps T2SS in *S. maltophilia* pathogenesis.

Recently, *S. maltophilia* has also been found to encode a VirB/VirD4 type IVA secretion system (T4SS) that is highly conserved within the species [60,61]. T4SSs are diverse systems found in both Gram-positive and Gram-negative bacteria, functioning to deliver DNA and/or effector proteins into eukaryotic or bacterial targets [62]. The *S. maltophilia* T4SS was found to promote both an antiapoptotic effect in lung epithelial cells as well as a proapoptotic effect on macrophages in a contact-dependent manner, likely due to the secretion of different effector proteins [60]. This system was also shown to give *S. maltophilia* a competitive growth advantage against other bacteria, including *Escherichia coli*, *Klebsiella pneumoniae*, and *P. aeruginosa*, due to the targeted bacterial cell killing through the secretion of effector toxins [60,61]. The role of the *S. maltophilia* T4SS in establishing infections as well as interacting with other pathogens in coinfections warrants further investigation.

Regulation of the expression of numerous *S. maltophilia* virulence factors is in part controlled by quorum sensing via the diffusible signal factor (DSF) system. First described in the related bacterial species *Xanthomonas campestris* pv. *campestris* as a regulator of virulence [63], research shows that the DSF quorum sensing system also controls many virulence-related phenotypes in *S. maltophilia* [64,65,66,67]. Stimulated RpfF synthesizes DSFs such as cis-Δ2-11-methyl-2- dodecenoic acid that is released to the extracellular environment; the sensor kinase RpfC detects accumulated DSF and induces the cytoplasmic regulator RpfG to degrade cyclic diguanylate monophosphate (c-di-GMP) to GMP, activating the transcriptional regulator Clp to stimulate virulence gene expression [68] (Figure 2). The effects of deletion of *rpfF* in *S. maltophilia* K279a and resultant loss of DSF production are pleiotropic, causing reductions in virulence and motility and changes in biofilm formation [64]. These effects could be reversed with *rpfF* complementation in trans or supplementation with DSF. In addition, DSF can stimulate the production and secretion of outer membrane vesicles found to contain the putative quorum sensing factor Ax21 among other proteins [69,70]. *S. maltophilia* secreted outer membrane vesicles are shown to have cytotoxic effects on human lung epithelial cells, stimulating the expression of proinflammatory cytokines and chemokines [71]. The putative diffusible signal Ax21 also has pleiotropic effects, with Ax21-deficient mutants exhibiting decreased motility, biofilm formation, tolerance to tobramycin and virulence compared to their wildtype counterparts [72]. Additionally, Ax21 abundance was shown to be directly proportional to mortality in a zebrafish model [44]. Motility deficits could be restored to wildtype levels in the presence of exogenous Ax21, consistent with the putative function as a signal molecule involved in cell-to-cell communication [72], however, due to the controversial research history of this protein [68], further investigation is needed.

As described above, cross talk between *S. maltophilia* and *P. aeruginosa* quorum sensing systems has significant implications for the clinical outcome of cystic fibrosis patients that have polymicrobial infections [37], therefore quorum quenching remains a strong therapeutic potential for further research. Although *S. maltophilia* isolates have the genetic potential for numerous virulence mechanisms, more research on the functionality of many of these virulence factors beyond homology relationships is needed to understand their role in *S. maltophilia* pathogenicity.

### 2.3. Antimicrobial Resistance Mechanisms of S. maltophilia

The rise in *S. maltophilia* infections worldwide is largely due to its intrinsic resistance to numerous frontline antibiotics. *S. maltophilia* exhibits resistance to a wide range of structurally unrelated antibiotics, including β-lactam antibiotics, macrolides, cephalosporins, aminoglycosides, carbapenems, chloramphenicol, tetracyclines, and polymyxins [3]. This resistance is attributed to multiple intrinsic and acquired antibiotic resistance mechanisms including reduced membrane permeability, numerous chromosomally encoded efflux pumps, β-lactamases, and aminoglycoside-modifying enzymes (Figure 2).

Typical of Gram-negative bacteria, *S. maltophilia* isolates exhibit reduced membrane permeability due to the rigid structure of their cell envelope that provides protection against the passive diffusion of antibiotics [73]. A major contributor to the high level of multidrug resistance observed in *S. maltophilia* strains is the numerous efflux pumps that mediate the active extrusion of multiple classes of antimicrobials across the largely impenetrable cell envelope. Multidrug efflux pumps form a tripartite double membrane-spanning channel consisting of an inner membrane protein that binds the substrate, an outer membrane porin, and a membrane fusion protein that connects the inner and outer membrane proteins in the periplasmic space [74]. Over a dozen efflux pumps have been identified in *S. maltophilia*, with the majority belonging to the resistance-nodulation-cell-division (RND) family [43]. These include SmeABC [75], SmeDEF [76,77], SmeGH [78], SmeJK [79], SmeMN [43], SmeOP [80], SmeVWX [81] and SmeYZ [82], the molecular mechanism for each characterized, with the exception of SmeMN. Two ATP binding cassette (ABC) family efflux pumps, SmrA [83] and MacABCsm [84], and one major facilitator superfamily (MFS) efflux pump, EmrCABsm [85] have also been characterized in *S. maltophilia*. The final efflux pump identified in this bacterium is FuaABC and contributes to fusaric acid resistance [86]. Collectively, these efflux pumps provide intrinsic and adaptive resistance to the antibiotics listed above [19,87].

Antimicrobial resistance in *S. maltophilia* is more elegant than the simple upregulation of efflux pumps. These bacteria encode a plethora of drug resistance mechanisms targeted to specific classes of antibiotics, many of which are antibiotic modifying enzymes. Resistance to β-lactam antibiotics in *S. maltophilia* is largely determined by two chromosomally encoded inducible β-lactamases, L1 and L2 [3,19,88]. L1 is a broad spectrum Zn^2+^-dependent metallo-β-lactamase and L2 is a clavulanic acid-sensitive cephalosporinase, both of which are regulated by AmpR, a transcriptional regulator located upstream of L2 [88]. The presence of a TEM-type β-lactamase encoded on a mobilizable Tn*1*-like transposon has also been reported in the genomes of clinical isolates of *S. maltophilia* [89]. Aminoglycoside resistance is primarily due to aminoglycoside-modifying enzymes, in addition to the efflux pumps SmeABC, SmeOP, SmeYZ, and MacABCsm in *S. maltophilia* [19]. Three of these enzymes have been characterized in *S. maltophilia* to date. These include the aminoglycoside acetyltransferases AAC(6′)-Iz [90] and AAC(6′)-Iak [91], and the aminoglycoside phosphotransferase APH(3′)-IIc [92].

Unlike other bacteria, quinolone resistant *S. maltophilia* isolates do not carry mutations in their topoisomerases [93]. Instead, low level resistance to quinolones stems from a chromosomal resistance gene, *smqnr*, encoding a pentapeptide repeat protein that protects the DNA gyrase and topoisomerase IV from inhibition by quinolones [94,95]. Additional quinolone resistance is largely provided by efflux pumps, including SmeDEF and SmeVWX [96,97]. The current recommended treatment for *S. maltophilia* infections is trimethoprim/sulfamethoxazole (TMP/SMX), and although susceptibility remains high, resistance to this antibiotic is increasing [98]. This is due to the presence of the sulfonamide resistance genes *sul1* carried by class 1 integrons and *sul2* associated with insertion sequence common region (ISCR) elements [25,99,100]. Dihydrofolate reductase encoding *dfrA* genes have also been found in Class 1 integrons and contribute to increased TMP/SMX resistance [98]. Additional TMP/SMX resistance in *S. maltophilia* is attributed to the efflux pumps SmeDEF, SmeOP, and SmeYZ. The choice of TMP/SMX as the recommended frontline treatment for *S. maltophilia* infections is also problematic due to potential sulfonamide allergies in patients and cross-reactivity with other drugs limiting its use [101].

The majority of antimicrobial resistance genes in *S. maltophilia* are not associated with mobile genetic elements, however intrinsic resistance via multidrug efflux pumps and aminoglycoside-modifying enzymes are widespread, with several families of efflux pumps ubiquitously present in *S. maltophilia* strains of all 23 lineages identified by Gröschel et al. [21]. The inability to control *S. maltophilia* infections due to this intrinsic and adaptive multi-drug resistance as well as a range of virulence factors increases mortality and morbidity and exemplifies the need for alternative treatments to combat this antibiotic resistant bacterium.

## 3. Phage Therapy

### 3.1. Bacteriophages

The pathogenicity and prevalence of *S. maltophilia* infections worldwide combined with the high level of antimicrobial resistance in these bacteria emphasizes the need for alternative treatments. Phage therapy is one promising treatment option under development. Bacteriophages, or phages for short, are bacterial viruses that recognize and bind to a specific host bacterium by recognition of a cell surface receptor to infect and kill the target bacterial species. Discovered over a century ago, phages were first used therapeutically to treat bacterial dysentery and cholera [102,103], however, controversy surrounding the efficacy of phage therapy combined with the discovery of broad-spectrum antibiotics in the 1940s meant that phages were no longer considered a viable treatment option in the West [104]. Research and application of phage therapy continued in Eastern European countries, however, with active phage therapy treatment centers such as the Eliava Institute in Tbilisi, Georgia and the Ludwik Hirszfeld Institute in Wrocław, Poland existing to this day [102,103].

Most phages undergo one of two replication cycles within a bacterial host cell following attachment of the viral particle to the bacterial cell surface [105]. Virulent phages replicate via the lytic cycle; the phage injects its genetic material into the cytoplasm and hijacks host cell metabolism to express phage genes and replicate its genome, followed by assembly of progeny virions that are released into the surrounding environment after phage-induced cell lysis. A successful infection by a virulent phage will release tens to hundreds of progeny phages that can infect surrounding bacterial cells, leading to exponential propagation. In contrast, temperate phages are capable of lysogeny, in which the phage genome integrates into the bacterial chromosome as a stable prophage or exists as a circular “phagemid” and lays dormant, replicating with the bacterial genome and passing vertically to daughter cells through bacterial cell division. In response to host cell stress or environmental cues, the prophage excises from the bacterial chromosome and resumes the lytic cycle to release progeny virions from the cell.

**Figure 3:**
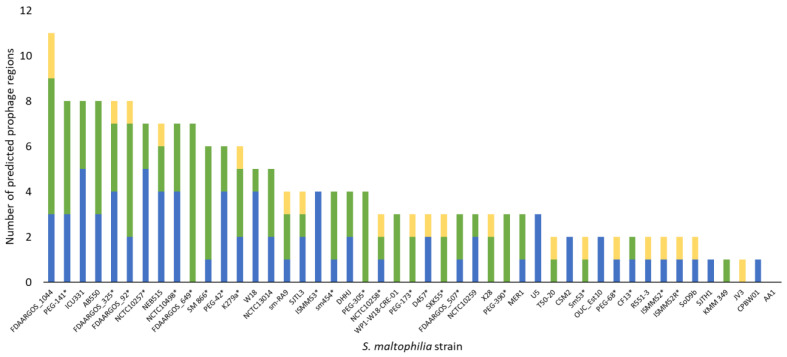
Prophage prevalence in 47 complete *S. maltophilia* genomes. Stacked bar graph showing the number of predicted prophage regions present in each *S. maltophilia* genome ranging from zero to eleven as determined by an updated version of PHAST [109,110]. Prophage regions are classified as intact (blue), incomplete (green), or questionable (yellow). Strains with * are clinical isolates and the remainder are environmental isolates, with the exception of FDAARGOS_1044, ICU331, NCTC13014, and NCTC10259 that are of unknown origin.

Prophages naturally exist in approximately half of sequenced bacteria, with many strains containing multiple intact or partial prophages that can constitute 10–20% of a bacterial genome [106,107,108]. To determine the prevalence of intact prophages and prophage remnants in *S. maltophilia* specifically, an updated version of PHAST [109,110] was used to identify putative prophage regions present in sequenced *S. maltophilia* strains with complete genomes in the NCBI database (February 2021). Of these 47 isolates, 23 are of clinical origin, 20 are from environmental sources and four are of unknown origin. Within the 47 unique genomes analyzed, 188 prophage regions were identified with 78 predicted to be intact prophages (Figure 3). Strain FDAARGOS_1044 (accession: NZ_CP065965.1) was predicted to have the most prophage regions with 11, three of which were classified as intact, six as incomplete, and two as questionable, whereas only one strain, AA1 (accession: NZ_CP018756.1), had zero predicted prophage regions. Although many bacteria have phage defense systems to protect against phage predation and possibly prophage integration, these systems, including CRISPR-Cas immunity, have not yet been characterized in *S. maltophilia* [111,112,113], potentially contributing to the high prevalence of prophage DNA in their genomes.

The high prevalence of prophages in bacterial genomes suggests that phages have played an important role in bacterial evolution [108,114]. Prophages may alter cell physiology and manipulate host metabolism by introducing new DNA that encodes novel functions. The integration of prophages affects the architecture of the bacterial genome and can account for a large portion of strain-to-strain genetic variation within a single species and in many cases, this contributes to the fitness of bacterial pathogens such as *Streptococcus pyogenes* [114,115] and shiga-toxin producing *E. coli* [116,117]. Temperate phages encoding accessory genes, or moron genes, can increase the host virulence or resistance to antibiotics during the lysogenic state, known as lysogenic conversion [114,118]. Additionally, prophages may transfer genes between bacteria by specialized transduction, potentially spreading antibiotic resistance or increasing bacterial virulence. Due to this, temperate phages are not considered as therapeutic candidates, however with advances in genetic techniques, discussed further below, these highly abundant temperate phages may be engineered to become suitable for therapeutic use.

There are numerous benefits to using phages therapeutically over antibiotics. As the most abundant biological entity in the biosphere at an estimated 10^31^ particles [119], phages are naturally occurring in the environment and therefore may be easily isolated for characterization. The majority of phages isolated from the environment using the current techniques are tailed phages belonging to the order *Caudovirales*, the biology of which is well understood [120,121]. Unlike broad spectrum antibiotics, phages are specific to a single bacterial species, due largely to the recognition of specific bacterial surface receptors. The use of phage therapy, therefore, does not harm beneficial bacterial flora or impose the risk of secondary *Clostridium difficile* bacterial infections due to depletion of the patient’s natural microbiome as observed following antibiotic treatment [122]. The specificity of phages can also be viewed as a negative due to the time needed to find a phage active against a specific strain, however, with advances in genetic engineering, the construction of broad host range phages is possible [123,124]. Phages have also recently been found to play a role in the structure and function of a healthy gut microbiome [125,126], with an estimated 31 billion phages transcytosed across the epithelial cell layers of the gut each day [127], and elicit limited or no host immune response [121]. Finally, the mechanism of action in phages differs from antibiotics, making phages effective against multidrug resistant bacteria, and the production of phage enzymes such as exo-polymer depolymerases allows phages to penetrate bacterial biofilms that inherently have increased drug resistance [122].

### 3.2. Clinical Data Using Phages

The rising antibiotic resistance crisis has led to increased interest in phage therapy in North America. In the last 15 years, nearly a dozen human clinical trials have been conducted to test the safety and efficacy of phages against numerous pathogens [128]. These trials have included single phage treatments as well as cocktails against priority pathogens, including *P. aeruginosa*, *Staphylococcus aureus,* and *E. coli*. The majority of trials administered phages topically at the site of infection or orally, however intraoperative and intravenous routes were also used. Overall, the outcomes from these trials suggest that phage therapy is tolerable, as few adverse effects were reported, however, the data from these trials are limited.

We have observed an increase not only in the number of approved phage therapy clinical trials in recent years but also in the number of compassionate use single patient cases treated with phages under expanded access Investigational New Drug (eIND) applications in the United States [129]. This is largely due to the creation of the first phage therapy center in North America in 2018, the Center for Innovative Phage Applications and Therapeutics (IPATH) affiliated with the University of San Diego School of Medicine in California, USA, and its role in helping patients access phage therapy. A review of the first ten cases of phage therapy conducted by IPATH revealed the safety and feasibility of intravenous phage treatment for a number of bacterial species and infection sites [129]. Adverse effects were rarely observed following phage administration and phages were successful in treating eight out of the ten patients; however, all patients were treated simultaneously with antibiotics, making it difficult to determine the effectiveness of phage treatment alone. These personalized phage therapy case studies have provided valuable empirical data and while the clinical data on phage therapy to date is promising, more translational research and controlled trials are needed.

For a comprehensive overview of the most recent compassionate use case reports and clinical data on phage therapy, see reviews by Luong et al. and Aslam et al. [128,129]. Additionally, Chan et al. summarize the therapeutic use of phages in cystic fibrosis cases specifically [130]. It should be noted that no human cases to date have included phage treatment for *S. maltophilia* infections.

### 3.3. Phage Therapy Strategies

Recent research has sought to determine strategies for effective delivery of phages to the site of infection, as well as combat challenges of administering phages. Although there are many options for the delivery of phages, such as inhalation, topical application, and intravenous injection, there are potential complications surrounding phage penetration of tissues and the inactivation of phage particles due to protein instability or clearance by the immune system [128,131,132].

To address this, researchers are investigating encapsulation of phages within a protective polymer or lipid matrices that increase phage stability and retention and can allow controlled release in vivo [131,132,133]. Encapsulation of phages provides a protective barrier, allowing phage particles to withstand adverse storage and physiological conditions, and penetrate deeper in the body while allowing controlled release at the site of infection [131,133]. Using pH-responsive microencapsulation of *E. coli* bacteriophages, Vinner et al. [134] show phage protection against the gastric acid environment of the stomach and effective release of phages at higher pH, as found in the intestine, while maintaining effective lytic ability against actively growing *E. coli*. Additionally, entrapment of phages within liposomes has been shown to provide 100% protection against phage neutralizing antibodies and maintain the killing ability of the phages against *K. pneumoniae* in vitro as well as within macrophages, demonstrating the potential to treat intracellular pathogens [135]. Further study using a liposomal entrapped phage cocktail to treat *K. pneumoniae* in a murine burn model showed increased phage retention time in vivo resulting in increased efficacy compared to free phage [136]. This research helps overcome major manufacturing, formulation, and delivery challenges of phage therapy.

Beyond delivery of phages to the site of infection, one of the major obstacles to developing effective phage therapies is the evolution of phage resistance arising in the bacterial host during the course of treatment. To overcome this, researchers suggest the use of phage cocktails that combine multiple phage isolates targeting different surface receptors to reduce the likelihood and speed of phage resistance evolving in a population, rather than single phage treatments [137]. These carefully designed phage mixtures decrease the risk of resistance arising to all phages in the mixture and broaden the lytic spectrum of a single treatment to target multiple bacterial strains, or in some cases species. For example, Yang et al. [138] designed a phage cocktail that is effective against a broad panel of *P. aeruginosa* clinical isolates using phages that target full-length and truncated O-antigen mutants, effectively constraining the emergence of phage resistance observed when using the phages individually. A similar approach to intelligent phage cocktail design was used against *Acinetobacter baumannii* with a mixture of phages that bind to both capsulated and uncapsulated cells to effectively control emergent resistant mutants [139]. The application of phages in combination with selected antibiotics can also increase the production and/or killing activity of phages, a phenomenon termed phage antibiotic synergy (PAS) [140]. Specifically, subinhibitory concentrations of antibiotics that lead to changes in cell morphology due to affected cell wall synthesis and cell division have been shown to increase the activity of phages targeting *E. coli* [140], *Burkholderia cepacia* [141], and *P. aeruginosa* [142], and combination phage and antibiotic treatments led to decreased mortality in a *Galleria mellonella* model [141] and increased biofilm clearance [142] compared to phage treatment alone. These examples demonstrate that carefully designed combinations of phages alone or together with antibiotics can increase the efficacy of phage therapy.

Another strategy is to harness the inevitable phage resistance that will arise by driving the evolution of a less fit bacterial population. Termed an anti-virulence strategy or phage steering [143,144,145], the use of phages that bind bacterial surface proteins that are important to pathogenicity or colonization of a host, such as pili, flagella, LPS, or capsule, can select for reduced virulence of the bacterial host due to mutation of the phage surface receptor. In addition to selecting for decreased bacterial virulence, surface receptor mutations in response to phage predation can also re-sensitize multidrug resistant bacteria to antibiotics. Recent research has shown that loss of capsule in *A. baumannii* in response to phage pressure not only decreased the virulence of resistant mutants, but also led to susceptibility to the human complement system, beta-lactam antibiotics, and phages with non-capsule receptors [146]. Similarly, a phage targeting the outer membrane protein of a *P. aeruginosa* multidrug efflux pump produced an evolutionary trade-off whereby phage resistance resulted in increased sensitivity to several classes of antibiotics [147]; this phage was later used in combination with antibiotics to successfully treat a patient’s life-threatening aortic graft infection [148]. Alternatively, phages may encode proteins that directly affect host cell virulence, such as the Tip protein of *Pseudomonas* phage D3112 that inhibits type IV pili-mediated twitching motility through interaction with the ATPase required for pili assembly [149]. A new family of small c-di-GMP interfering peptides known as YIPs has also been identified in PB1-like *Pseudomonas* phages that affect c-di-GMP regulated virulence processes such as motility and biofilm formation [150]. Intelligent design of cocktails containing phages that interfere with or bind important virulence factors or antimicrobial resistance proteins shows great promise as a strategy, as phage resistant mutants that arise will be more susceptible to secondary antimicrobial treatments and clearance by the immune system.

### 3.4. S. maltophilia Phages

In 1973, when *S. maltophilia* was believed to be a species of *Pseudomonas*, early research investigated phages as genetic tools to study the genetic maps of different species of *Pseudomonas* using transduction [151]. The first isolated *S. maltophilia* phage, M6, was induced as a prophage from *P. maltophilia* strain 6 and was capable of infecting four out of the 50 *P. maltophilia* strains tested [151]. A host range mutant, M6a, was isolated by plating high titre M6 lysate on *P. aeruginosa* and further study revealed that it is a general transducing phage, however, this variant was unable to infect the original *P. maltophilia* hosts, therefore interspecies transductions were unable to be performed. No further research on phages as genetic tools for *S. maltophilia* has been described since phage M6.

In the 21st century, research on *S. maltophilia* phages shifted its focus to the isolation and characterization of phages for therapeutic applications. In 2005, Chang et al. isolated eight *S. maltophilia* phages from clinical samples, including patient specimens and catheter-related devices, and wastewater samples [152]. These phages were divided into four groups based on host range analysis using a panel of ten strains and a single phage, ΦSMA5, was chosen for further characterization. ΦSMA5 is a broad host range phage and exhibits a large burst size, producing approximately 95 virions per cell. Analysis of viral DNA by restriction fragment length polymorphism (RFLP) revealed possible modifications to A and/or T bases based on the observed resistance to digestion, and although genome sequencing was not completed, pulse-field gel electrophoresis (PFGE) suggested ΦSMA5 has a large genome approximately 250 kb in size. No data on phage lifestyle was described, however, the authors refer to ΦSMA5 as a virulent phage in future publications [153,154]. In 2007 the same group of researchers published their characterization of Smp14, a T4-like virulent phage isolated from hospital sewage in Taiwan [153]. Electron microscopy revealed a *Myoviridae* morphology and phage particles were observed binding to the poles of the host cells. No receptor was identified, however, based on their observations it is likely that Smp14 interacts with polar structures such as the flagella or type IV pili that may have been retracted during imaging. Functional analyses show that phage Smp14 infects only 37 out of 87 strains, however, compared to phage ΦSMA5, Smp14 has a faster adsorption rate, shorter latent period, and larger burst size, with roughly 150 progeny released per cell [153]. Characterization of the roughly 160 kb phage genome revealed the presence of modified bases resulting in resistance to digestion by many restriction enzymes. Partial sequencing of a 16 kb region containing 14 ORFs predicted to encode structural proteins showed 15–45% identity to structural proteins of T4-like phages as well as similar organization, classifying Smp14 as a T4-like phage [153]. Based on its virulent lifestyle and infection dynamics, the authors suggest that this phage has potential for inclusion in phage cocktails for the treatment of *S. maltophilia* infections.

The following year, Garcia et al. isolated 22 phages against *S. maltophilia* from sewage and prophage induction, and the three phages with the broadest host ranges were chosen for further study [155]. The temperate *Siphoviridae* phage S1 was induced from an environmental *S. maltophilia* strain and has a narrow host range. Phages S3 and S4 were isolated from sewage samples and analysis of their genomic DNA suggested the presence of abnormal bases due to the resistance to restriction digestion. S4 was also identified as a temperate *Siphoviridae* phage with a broader host range than S1. Phage S3 belongs to the *Myoviridae* family and is a presumed virulent phage based on the low frequency of phage resistant mutants isolated compared with temperate phages S1 and S4. Based on these properties in addition to the large burst size of approximately 100 phages produced per infected cell, the authors suggest that S3 is a candidate for therapeutic application [155]. However, further analysis, including genome sequencing and confirmation of phage lifestyle, is required to assess the safety of S3.

Four years later, a group of researchers from Beijing published their characterizations of two virulent phages that they isolated from hospital sewage samples, IME13 [156] and IME15 [157]. In the absence of electron microscopy data, genome sequencing suggests that IME13 is a *Myoviridae* phage with a genome over 162 kb in size [156], while IME15 is a T7-like phage belonging to the family *Podoviridae* and has a genome 38.5 kb in size [157]. Interestingly, IME13 has an incredibly large burst size that exceeds 3000 phage produced per cell and has a unique plaque polymorphism, producing plaques of three different sizes [156]. Host range and infection dynamics were not described, however, the virulent nature and large burst sizes of these phages make them strong anti-*S. maltophilia* candidates.

In 2013, a translated abstract of a journal article published by a group of researchers from China described the first in vivo use of phage against *S. maltophilia* [158]. Phage SM1 was isolated from hospital sewage and is described as a relatively broad host range phage with a rapid infection cycle and large burst size of 187 virions per cell. Although lifestyle was not mentioned, the resistant mutation rate was low, at 10^−10^, suggesting SM1 may be virulent. Most notable, SM1 is effective in vivo; using a mouse infection model, all *S. maltophilia* infected mice survived to seven days post-infection when treated with SM1 phage [158].

In 2014, seven years after their publication on Smp14, Lee et al. described phage Smp131, a temperate *Myoviridae* phage isolated from the culture supernatant of *S. maltophilia* T13 [154]. Smp131 has a narrow host range and based on protein similarity and genomic organization of the 33.5 kb genome, Smp131 is classified as a P2-like phage similar to many prophages in *S. maltophilia* and *Xanthomonas* species. Interestingly, Smp131 encodes a novel phage endolysin similar to members of the GH19 chitinase family previously only identified in plants and bacteria.

The following year two remarkable virulent phages were discovered by our group that are capable of infecting across taxonomic orders, lysing not only *S. maltophilia* as their main host, but also strains of the nosocomial pathogen *P. aeruginosa* [11]. Phages DLP1 and DLP2 were isolated from soil samples and genomic characterization revealed genomes approximately 42 kb in size sharing 96.7% identity over 97.2% of their genomes, in addition to high genetic similarity to numerous *Pseudomonas* phages. Further study of DLP1 and DLP2 identified the host receptor as the type IV pilus across strains of both host genera [144]. This interaction can be observed in Figure 1, showing phage DLP1 particles binding to pili extending from the surface of *S. maltophilia* strain D1585. These virulent phages are promising candidates for phage therapy as the co-occurrence of *S. maltophilia* and *P. aeruginosa* is common in cystic fibrosis patient lungs [3,35].

In 2017, we published on the characterization of a third virulent phage, DLP6 [159]. Having a moderate host range, transmission electron microscopy and genome sequencing classified DLP6 as a divergent T4-like *Myoviridae* phage that contains genomic features from the T4-superfamily of both enteric phages and cyanophages. Also published in 2017 was a study using virulent phages to treat a corrosion-producing *S. maltophilia* strain isolated from a petroleum pipeline in Iran [160]. Unnamed phages were isolated from surrounding wastewater and electron microscopy showed a *Siphoviridae* morphology with unusually long tails over 400 nm long. Phage treatment reduced bacterial growth by 50% in vitro, however, no further information was provided. This study provides an example of the potential industrial application of *S. maltophilia* phages to treat biocorrosion in addition to human therapy.

Over the next three years, we described three additional temperate phages for *S. maltophilia* [112,161,162]. Genome sequencing of the *Siphoviridae* phage DLP5 revealed less than 2% identity with any phages in the NCBI database, leading to the establishment of the new genus *Delepquintavirus* [161]. This phage has a narrow host range, replicates as a phagemid during lysogeny, and is capable of lysogenic conversion of its host. Closely related to DLP5 is the *Siphoviridae* temperate phage DLP3, the second member of *Delepquintavirus* [162]. Despite genomic similarity to DLP5, phage DLP3 has a much broader host range, infecting 22 out of 29 clinical *S. maltophilia* strains, and was shown to use the type IV pilus as its receptor. DLP3 also causes lysogenic conversion of its host due to the expression of a functional erythromycin resistance protein as well as unknown factors that cause increased growth rate and virulence in a *G. mellonella* infection model compared to the wildtype non-lysogen. Despite this effect in the lysogen, we show that DLP3 is capable of rescuing *G. mellonella* larvae infected with *S. maltophilia* strain D1571 at an MOI of 100 with 53% of larvae surviving to 120 h compared to 17% survival in the untreated controls [162]. The third temperate phage we have characterized is the *Siphoviridae* phage DLP4 that is also capable of lysogenic conversion of its host due to expression of functional FolA and YbiA proteins involved in trimethoprim resistance and swarming motility, respectively [112]. No lysogeny related genes were identified. The researchers show that this phage also uses the type IV pilus for infection across its host range.

The final *S. maltophilia* phage paper published by our group characterizes a novel virulent *Siphoviridae* phage, AXL3 [113]. This phage has a narrow host range and long productive cycle, with approximately 38 phages released per cell after 6.5 h, and was identified to also use the type IV pilus as its host receptor. RFLP analysis revealed a restriction resistant genome with possible G and/or C base modifications and genome sequencing suggests that this phage may belong to a new genus based on limited identity to other phages in the NCBI database.

Four *S. maltophilia* phages were isolated by a group from the Center for Phage Technology (CPT) at Texas A&M University. Phages Ponderosa [163] and Pokken [164] are *Podoviridae* phages isolated from water samples and phages Moby [165] and Mendera [166] are *Myoviridae* phages isolated from wastewater. Complete genomic characterization for each phage is available on NCBI, however, no experimental data regarding host range, lifecycle, or phage infection dynamics were provided to evaluate the suitability of these phages for therapeutic use. Recently, a group of researchers from China published their characterization of the novel phage BUCT548, identifying it as a *Siphoviridae* phage with limited sequence identity to *Pseudomonas* phage PBPA162 [167]. Functional characterization shows that BUCT548 has a broad host range, short productive cycle, and large burst size, however, no analysis of phage lifestyle was completed.

The final *S. maltophilia* phage functionally characterized to date is ΦSHP3, a B3-like transposable *Siphoviridae* phage with a small genome of 37.6 kb [168]. In addition to the conserved genes Mor, GemA, TnpA, and TnpB widely distributed in transposable phages, ΦSHP3 also encodes a functional RdgC exonuclease protein that possibly plays a role in phage recombination. Investigation into the regulation of lytic-lysogenic switch suggested that the SOS response may play a role due to the presence of LexA and CpxR binding motifs [168]. Further characterization of ΦSHP3 as the first transposable phage of *S. maltophilia* will provide information on the role phages play in the genetic heterogeneity of *S. maltophilia* and may become a powerful tool for genetic manipulation in this species.

A search of NCBI revealed four additional genomes for *S. maltophilia* phages isolated in China and the USA with no corresponding publications, therefore morphology and lifestyle may only be speculated based on genomic content. Phages IME-SM1 (accession: KR560069) and YB07 (accession: MK580972) are closely related phages with genomes approximately 160 kb in size of the family *Ackermannviridae*. Phage Salva (accession: MW393850) was isolated from soil and is closely related to the *Siphoviridae* phage BUCT548, also encoding 102 ORFs and one tRNA in its approximately 60 kb genome. Finally, phage BUCT555 (accession: MW291508) is a novel *Podoviridae* phage with a genome nearly 40 kb in size having no close relatives in the NCBI database.

While not useful for therapeutic applications, numerous filamentous phages have also been described in *S. maltophilia.* Filamentous phages in the family *Inoviridae* are characterized by their unique morphology, small single-stranded DNA genomes, and chronic infection cycle whereby progeny virions are continuously released without killing the host. In 2006, Hagemann et al. discovered a self-replicating DNA molecule in genome preparations of a clinical *S. maltophilia* strain [169]. Sequencing of the extra-chromosomal DNA molecule revealed a 6709 bp linear genome encoding seven proteins, including a putative Zonula occludens-like toxin (zot) with sequence identity to the Zot toxins of *Xylella* and *Vibrio cholerae*. The authors named this novel filamentous phage ΦSMA9 based on the resemblance of the size and gene organization of the DNA molecule to the replicative form of other filamentous phages [169]. Several filamentous phages have since been identified in environmental *S. maltophilia* isolates. In 2012 and 2013, a second group detailed their findings of the novel *Inoviridae* phages ΦSHP1 and ΦSHP2 isolated from the environmental *S. maltophilia* strains P2 and P28 [170,171]. Electron microscopy of ΦSHP1 showed filamentous structures approximately 2.1 μm long. Sequencing of the 6867 bp genome previously isolated in its replicative form as pSH1 revealed ten putative ORFs including a predicted Zot-like toxin [170]. Electron microscopy of ΦSHP2 revealed filamentous particles 0.8 μm long that contained single-stranded DNA [171]. Sequencing of the replicative form, pSH2, revealed similarities to ΦSHP1 and ΦSMA9, including a Zot-like toxin gene present in the 5819 bp length genome. Two additional filamentous phages were identified in 2014 from an environmental isolate, *S. maltophilia* strain Khak84, as extrachromosomal genetic elements [172]. Sequencing revealed two contigs approximately 7 kb in size with similar gene organization and homology to other filamentous *Inoviridae* phages. Both genomes encode 11 putative ORFS, including zot-like genes. Recent investigation of microbial genomic sequencing data has identified a vast heterogeneity and widespread distribution of *Inoviridae* phages that were previously underappreciated [173]. Filamentous phages are abundant in other human pathogens, such as *P. aeruginosa* [174], and have been shown to increase the virulence of their bacterial host and interact with the human immune system during infection [175,176], prompting concern into the role of filamentous phages in *S. maltophilia* pathogenicity. Although all five filamentous phages identified in *S. maltophilia* strains to date encode a Zot-like protein, the sequences are divergent and further research is needed to determine the functionality of these proteins as toxins and whether they affect host virulence.

In addition to harnessing active phages for their antimicrobial properties, individual phage proteins have also been characterized for use against antibiotic resistant bacteria, including *S. maltophilia*. Phages encode enzymes called endolysins or lysozymes that degrade the peptidoglycan of the bacterial cell wall from within during the final stage of the phage lytic replication cycle, causing host cell lysis [177]. Research has shown that endolysins can also be effective when applied externally to the cell. In 2006, Lee and colleagues characterized a *Xanthomonas oryzae* phage lysozyme, Lys411, and found it active against not only its host species, but it also had even stronger activity against *S. maltophilia* [178]. The number of *S. maltophilia* strains Lys411 is active against was not indicated and no follow-up studies have been published, meaning the potential of this enzyme for therapeutic control of *S. maltophilia* infections is unknown. Bacterial genomes may also carry gene clusters that encode phage tail-like bacteriocins (PTLBs). These large protein complexes resemble the tails of *Siphoviridae* and *Myoviridae* phage particles and have bactericidal activity against bacteria related to the producing strain [179]. Two PTLBs have been identified in *S. maltophilia*, maltocin P28 and S16 [171,180]. Liu and colleagues identified maltocin P28 as phage tail-like particles in electron micrographs of filamentous phage ΦSHP2; purification of these particles indicated that they contained no genetic material but had antimicrobial activity against both environmental and clinical *S. maltophilia* isolates [171]. In 2019, the same group of researchers published on a second maltocin, S16, that had broad antibacterial activity against 62 out of 86 *S. maltophilia* strains tested and remarkably eight out of 14 *E. coli* strains [180]. The authors suggest that maltocins are widespread in *S. maltophilia*, possibly providing a range of novel antimicrobial alternatives to antibiotics yet to be discovered.

**Figure 4:**
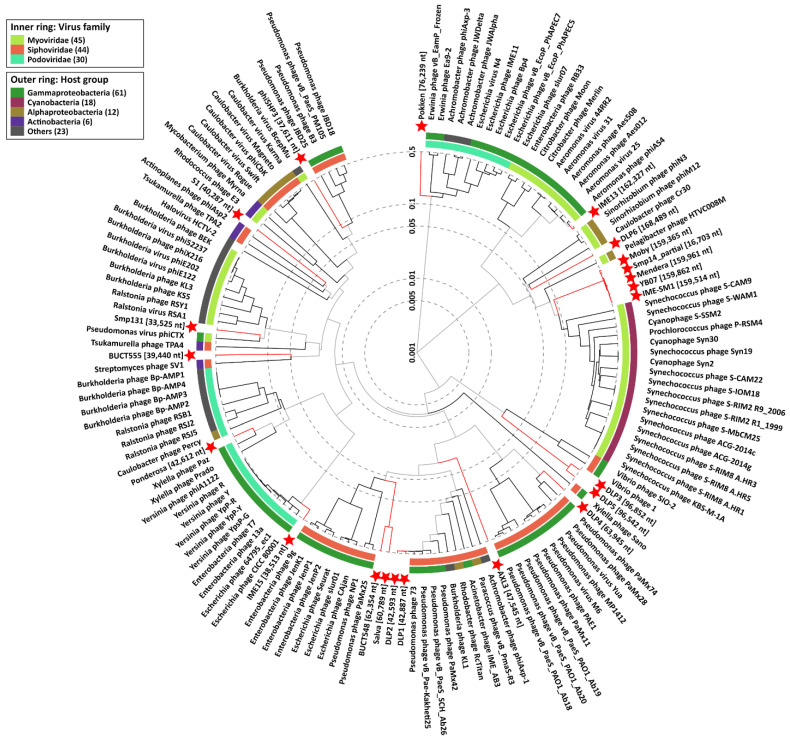
*S. maltophilia* phage phylogenetic tree. The results of ViPTree analysis using a protein distance metric based on normalized tBLASTx scores plotted on a log scale. The tree includes 142 dsDNA phages with the 22 *S. maltophilia* phages with genome sequencing data highlighted with red stars. Related phages chosen for inclusion were selected as the top ten phages with the highest genome similarity S_G_ scores for each of the 22 *S. maltophilia* phages with sequencing data available. This tree was generated using the ViPTree server [181].

In summary, 32 phages have been isolated and characterized against *S. maltophilia*, with their key features described in Table 1. Five of these phages belong to the family *Inoviridae*, each encoding a putative Zot-like toxin, and are not useful for therapy, however they may play ecological roles and influence the pathogenicity of their host. Of the 27 double-stranded DNA phages, 22 have genome sequencing data available. Phylogenetic analysis of these phage proteomes using ViPTree [181] shows the extreme diversity found within the *S. maltophilia* phages isolated to date (Figure 4). Five of the phages, IME-SM1, YB07, Mendera, Smp14, and Moby, group together in the same clade and represent many of the T4-like *S. maltophilia* phages, with the exception of DLP6. Similarly, the T7-like phages Ponderosa and IME15 share limited protein similarity with each other; these examples highlight the diversity present with the T4-like and T7-like groups of phages. Apart from the relationships between DLP5 and DLP3, DLP1 and DLP2, and BUCT548 and Salva, the remaining phages share low protein sequence similarity with each other as well as with phages infecting other bacterial species, and phages such as AXL3 and S1 may even belong to new genera. The extreme diversity within *S. maltophilia* phages is promising for the creation of effective broad-range phage cocktails, as well as the study of novel phage biology mechanisms.

### 3.5. Potential of Phage Therapy for S. maltophilia

Of the *S. maltophilia* phages with experimentally confirmed lifecycles, nine are virulent and potentially desirable for therapeutic use, while eight are temperate and capable of lysogeny, and in the case of phage ΦSHP3, transposition. For some phages with genomic sequencing and characterization, lifestyle was not determined, which is essential prior to use in therapy. The nine virulent phages, ΦSMA5, Smp14, S3, IME13, IME15, DLP1, DLP2, DLP6, and AXL3, isolated and characterized for their potential use in phage therapy are diverse (Figure 4).

Interestingly, five of the isolated phages were shown to use the same receptor for host recognition; isolated from soil, phages DLP1 (Figure 1), DLP2, DLP3, DLP4, and AXL3 all require a functional type IV pilus capable of retraction for successful infection. The cell surface receptor was not determined for the remaining phages, a seemingly overlooked aspect in phage characterization [137]. While the type IV pilus has not yet been well characterized in *S. maltophilia*, the assembly machinery and pili structure is highly conserved across bacteria and has been extensively characterized in the bacterial pathogens *P. aeruginosa* and *Neisseria* spp. as an important virulence factor [53,182]. Mutational studies in these bacteria show that loss of type IV pili results in a decreased ability to form biofilms and decreased virulence in vivo [183,184]. The apparent favoring of the type IV pilus as a receptor for *S. maltophilia* phages suggests that this structure plays an important role in bacterial survival and likely virulence, as observed in other pathogens. Therefore, the use of phages that target the pilus are ideal candidates for anti-virulence therapeutics; should phage resistance arise due to modification or loss of the pilus, these mutants will have obtained phage resistance at the cost of lowered virulence and fitness in a host. Although identification of phage receptors can be time consuming, characterizing the additional receptors for *S. maltophilia* phages will inform their use as therapeutics in effective phage cocktails, emphasizing the need for routine receptor identification.

The even distribution of phage replication types and wide range of isolation sources is promising for the discovery of additional virulent *S. maltophilia* phages to be included in phage cocktails for therapy. However, compared to the over 400 phage genomes available in NCBI against the nosocomial pathogen *P. aeruginosa,* more phages must be isolated against *S. maltophilia* to make phage therapy a more feasible treatment option for multidrug resistant infections caused by this heterogeneous bacterium.

### 3.6. Perspectives and Future Directions

Due to the heterogeneity of *S. maltophilia* isolates, treatment of infections will require diverse cocktails of phages and combination therapy with antibiotics or other phage-derived antimicrobials. While regulatory agencies and clinicians generally balk at the inclusion of temperate phages, properties such as the large burst size of S4 and broad host range of DLP3 make these phages desirable for therapeutic applications. Advances in sequencing technologies and synthetic biology may provide new opportunities to explore the use of modified versions of these phages for therapy by eliminating less favorable features or enhancing bacterial killing in different conditions and effectively improve the safety and efficacy of temperate phages [185]. The predicted abundance of prophages in *S. maltophilia* genomes (Figure 3), and bacterial genomes in general, described above makes finding and isolating temperate phages easier than virulent phages. Recently, a technique was described by Mageeney et al. [186] to mine bacterial genomes related to a target strain of interest for prophage elements that can be isolated and engineered to become lytic through the deletion of the integrase gene. The researchers show proof of concept using five prophages isolated from a single *P. aeruginosa* strain that they engineered for nonlysogeny and were effective against *P. aeruginosa* PA01 in both liquid and *G. mellonella* infection trials. This research sets the precedence to create a platform for on-demand production of therapeutic phages from closely related bacterial strains.

While engineering temperate phages into lytic variants is desirable, genetic modification of lytic phages can also overcome limitations and successfully expand phage host range, reduce toxicity and immunogenicity, and improve activity against biofilms or in combination with antibiotics [124,185,187]. A suite of genetic tools is available for the genetic modification of phages. Homologous recombination although common, is labor intensive and time consuming, while more efficient techniques such as bacteriophage recombineering of electroporated DNA (BRED) are limited to specific species, namely mycobacteriophages [187]. Most recently this technique was used to engineer a lytic derivative of a phage targeting *Mycobacterium abscessus* through deletion of its lytic repressor gene and was subsequently used in combination with two other phages to treat a patient with cystic fibrosis following bilateral lung transplantation in the United Kingdom who was suffering from an antibiotic resistant infection [188]. This was the first use of engineered phages in human therapy and spearheads the acceptance of genetically engineering phages for human treatment.

Perhaps more widely applicable is Gibson assembly, the construction of synthetic phage genomes from PCR amplified fragments, a technique used by Mageeney et al. to construct their lytic prophage variants [186]. Yeast-based assembly of synthetic phages from PCR fragments has also proved efficient for the modification of phage genomes [187]. Recently, Pires et al. used yeast recombineering to construct a minimal phage lacking numerous genes encoding hypothetical proteins that made up to 48% of its original genome with no deleterious effects on phage antibacterial efficacy [189]. Removal of genes encoding hypothetical proteins with unknown function creates room in the phage genome for replacement with genes encoding additional receptor binding proteins or enzymes to aid in host range expansion and cell lysis while remaining within the genome packaging capacity of the phage. However, care must be taken to ensure that hypothetical proteins chosen for removal do not negatively affect phage fitness. Additionally, phage hypothetical proteins encoded by early genes significantly affect host metabolism during phage infection and may have bactericidal effects upon overexpression, demonstrating an untapped source of inspiration for novel antimicrobial molecules with specific narrow spectrum bacterial targets [190,191].

Genome engineering of virulent phages has also been possibly using CRISPR-Cas9, with studies showing effective gene replacement and deletion in lytic phages of *Streptococcus thermophilus* [192] and *Lactococcus lactis* [193]. Recently, the FDA approved a clinical trial (NCT04191148) sponsored by Locus Bioscience to treat urinary tract infections with their genetically engineering crPhage cocktail, containing CRISPR Cas3-enhanced phages targeting *E. coli* and will be the first controlled clinical trial for recombinant bacteriophage therapy, paving the way for future studies.

Though the potential application of these techniques to phage therapy has been shown in other species, genetic engineering of *S. maltophilia* phages has yet to be described. Due to the trend of DNA modification observed in *S. maltophilia* phages as the inability of numerous restriction endonucleases to digest DNA (Table 1), targeted genetic manipulation techniques may prove difficult. For this reason, alternative methods may be explored for the modification of *S. maltophilia* phages without the need for direct molecular manipulation. Directed evolution approaches harness principles of the natural arms race between phage and bacteria that has occurred in nature for over three billion years [194]. In the “Appelmans protocol,” spontaneous mutation and recombination occur among a cocktail of phages grown together on a range of susceptible and resistant bacterial strains over several generations resulting in phages with expanded host ranges, created without the addition of new exogenous genetic information [194]. Chemically accelerated viral evolution (CAVE) is another approach to rapidly enhance desired phage characteristics through iterative cycling of chemical mutagenesis and phage selection [195]. Proof of principle was demonstrated using *E. coli* and *Salmonella enterica* phages evolved to possess improved thermal tolerance and stability over 30 rounds of mutagenesis and selection. The authors suggest that a variety of selection criteria may be employed using this platform to develop phages with increased therapeutic potential.

Beyond modification of *S. maltophilia* phages for therapy, reports of in vivo studies to determine the therapeutic potential of these phages are limited. Apart from rescue of *S. maltophilia* infected *G. mellonella* larvae by temperate phage DLP3 [162] and murine rescue by phage SM1 [158], the behavior and therapeutic potential of *S. maltophilia* phages are largely unknown. Testing the behavior of individual phages and combinations in animal models is essential to determine their initial efficacy for therapy, as some phages that exhibit strong therapeutic potential in vitro are ineffective or unstable during in vivo trials [196]. The pharmacokinetics and effectiveness of phages following aerosolization, intravenous injection, or topical applications that mimic treatment of *S. maltophilia* lung, bloodstream, and wound infections must also be explored.

## 4. Summary

With the prevalence of multidrug resistant *S. maltophilia* infections rising worldwide, particularly in the cystic fibrosis community, research into the mechanisms underlying disease progression and resistance to antimicrobials is essential. The frontline recommended antimicrobial drug of choice against *S. maltophilia* infections is TMP/SMX, however, resistance to this antibiotic is on the rise globally due to the spread of *sul* and *dfrA* genes [98]. With pharmaceutical companies largely abandoning the development of novel antibiotics due to a lack of return on investment [197,198], alternative therapeutics must be investigated to combat these multidrug resistant *S. maltophilia* infections.

Bacteriophages with the proper characterization represent a promising alternative treatment option for antimicrobial resistant bacterial infections. The isolation of 28 unique phages against *S. maltophilia* in the last 15 years, at least nine of which are virulent, demonstrates the ease of isolation and shows promise for the future application of phage therapy against this pathogen. Further in vivo research into the efficacy of these phages individually and in multi-phage cocktails or in combination with other antimicrobials is needed to spearhead the clinical use of *S. maltophilia* phage therapy.

## Figures and Tables

**Table 1:** *S. maltophilia* phage characteristics.

Phage	Source; Isolation Strain	Genome Length (bp)	GC (%)	Family	Phage Relatedness ^1^	Lifestyle	Unique Features	Reference; Accession If Applicable
M6	*P. maltophilia*^a^ 6	–	–	*Siphoviridae*	–	Temperate	First phage isolated for *S. maltophilia* Transducing phage Host range mutant, M6a, is capable of infecting *P. aeruginosa*	[151]
ΦSMA5	Sputum; *S. maltophilia* T39	~250 kb ^b^	–	*Myoviridae*	–	Virulent	Broad host range, 61 out of 87 strains susceptible Burst size 95 phages/cell DNA is restriction enzyme resistant	[152]
Smp14	Sewage; *S. maltophilia* T14	~160 kb ^c^	53.3 ^c^	*Myoviridae*	*Stenotrophomonas* phage YB07	Virulent	T4-like phage Moderate host range infecting 37 out of 87 clinical isolates Adsorbs to poles of cells Burst size ~150 phages/cell DNA is restriction enzyme resistant	[153] DQ364602
S1	Environmental *S. maltophilia* CECT 4793	40,287	63.7	*Siphoviridae*	<1% coverage to *Stenotrophomonas* phage Smp131	Temperate	Narrow tropism, infecting 4 out of 26 strains Encodes putative GspM protein involved in host type II secretion system Burst size of ~75 phages/cell 48 ORFs	[155] NC_011589
S3	Sewage; *S. maltophilia* E539	~33 kb ^b^	–	*Myoviridae*	–	Virulent	Moderate host range infecting 12 out of 26 strains Burst size ~100 phages/cell Short eclipse period of 30 min DNA is restriction enzyme resistant	[155]
S4	Sewage; *S. maltophilia* F227	~200 kb ^b^	–	*Siphoviridae*	–	Temperate	Broad host range infecting 18 out of 26 strains Burst size ~80 phages/cell DNA is restriction enzyme resistant	[155]
IME13	Sewage; clinical *S. maltophilia*	162,327	41.2	*Myoviridae* ^d^	>97% *Aeromonas* phage phiAS4	Virulent	Large burst size >3000 phages/cell Plaque polymorphism with three plaque sizes 182 ORFs; 15 tRNAs	[156] JX306041
IME15	Sewage; clinical *S. maltophilia*	38,513	53.7	*Podoviridae* ^d^	>97% *Aeromonas* phage PZL-Ah1	Virulent	T7-like phage Burst size >100 phages/cell 45 ORFs	[157] JX872508
SM1	Sewage; *S. maltophilia*	~50 kb ^b^	–	*Myoviridae*	–	–	Large burst size of 187 phages/cell In vivo mouse trials show 100% of SM1 treated mice surviving past day 7	[158]
Smp131	Clinical *S. maltophilia* T13	33,525	65.0	*Myoviridae*	Uncultured *Caudovirales* phage clone 3S_12	Temperate	P2-like phage Narrow tropism, infecting 3 out of 86 strains 47 ORFs	[154] JQ809663
DLP1	Red Deer River sediment; clinical *S. maltophilia* D1585	42,887	53.7	*Siphoviridae*	>97% to *P. aeruginosa* phage SCUT-S4	Virulent	Host range crosses taxonomic orders to *P. aeruginosa* strains. Uses type IV pili as host receptor 57 ORFs	[11,144] KR537872
DLP2	Blue flax soil; clinical *S. maltophilia* D1585	42,593	53.7	*Siphoviridae*	>97% to *P. aeruginosa* phage PA73	Virulent	Host range crosses taxonomic orders to *P. aeruginosa* strains. Uses type IV pili as host receptor 58 ORFs	[11,144] KR537871
DLP3	Empty soil; clinical *S. maltophilia* D1571	96,852	58.3	*Siphoviridae*	*Stenotrophomonas* phage DLP5	Temperate	Uses type IV pili as host receptor Second member of the *Delepquintavirus* genus Broad host range infecting 22 out of 29 strains Therapeutically active in D1571 infected *G. mellonella* larvae Causes lysogenic conversion of D1571 Encodes functional erythromycin resistance protein DNA is restriction enzyme resistant 148 ORFs; 5 tRNAs	[162] MT110073
DLP4	Planter soil; clinical *S. maltophilia* D1585	63,945	65.1	*Siphoviridae*	*Xanthomonas* phage Bosa	Temperate	Moderate host range infecting 14 out of 27 strains Uses type IV pili as host receptor Causes lysogenic conversion of host Encodes functional trimethoprim resistance protein and virulence factor YbiA DNA is restriction enzyme resistant 82 ORFs; 1 tRNA	[112] MG018224
DLP5	Empty soil; clinical *S. maltophilia* D1614	96,542	58.4	*Siphoviridae*	*Stenotrophomonas* phage DLP3	Temperate	Type strain of *Delepquintavirus* genus Temperate phage is maintained as a phagemid Narrow host range infecting 5 out of 27 strains Causes lysogenic conversion of D1614 Encodes putative erythromycin resistance protein DNA is restriction enzyme resistant 149 ORFs; 5 tRNAs	[161] NC_042082
DLP6	Planter soil; clinical *S. maltophilia* D1571	168,489	55.8	*Myoviridae*	*Sinorhizobium* phage phiN3	Virulent	Moderate host range infecting 13 out of 27 strains Divergent T4-like virus Encodes a transposase DNA is restriction enzyme resistant 241 ORFs; 30 tRNAs.	[159] KU682439
AXL3	Empty soil; clinical *S. maltophilia* D1585	47,545	63.3	*Siphoviridae*	4% coverage to *Pseudomonas* phage JG012	Virulent	Narrow host range infecting 5 out of 29 strains Uses type IV pili as host receptor Long infection cycle with burst size of 38 phages/cell DNA is restriction enzyme resistant 65 ORFs	[113] MT536174
Ponderosa	Water sample; *S. maltophilia* ATCC 17807	42,612	60.0	*Podoviridae*	*Xylella* phage Paz	–	T7-like phage 54 ORFs	[163] MK903280
Pokken	Water sample; *S. maltophilia* ATCC 17807	76,239	55.1	*Podoviridae*	*Xanthomonas* phage RiverRider	–	92 ORFs; 5 tRNAs	[164] MN062186
Moby	Wastewater; *S. maltophilia* ATCC 17807	159,365	54.1	*Myoviridae*	*Stenotrophomonas* phage Mendera	–	T4-like phage 271 ORFs; 24 tRNAs	[165] MN095772
Mendera	Wastewater; *S. maltophilia* ATCC 17807	159,961	54.0	*Myoviridae*	>97% to *Stenotrophomonas* phage YB07	–	T4-like phage 287 ORFs; 23 tRNAs	[166] MN098328
BUCT548	*S. maltophilia* 824	62,354	56.3	*Siphoviridae*	*Stenotrophomonas* phage Salva	–	Broad host range infecting 11 out of 13 strains Burst size 134 phages/cell 102 ORFs; 1 tRNA.	[167] MN937349
phiSHP3	*S. maltophilia* c31	37,611	65.3	*Siphoviridae*	*Pseudomonas* phage B3	Temperate	Transposable phage Moderate host range infecting 20 out of 83 strains 51 ORFs	[168] MT872956
IME-SM1	Hospital sewage	159,514	54.1	*Ackermannviridae*	>98% to *Stenotrophomonas* phage YB07	-	254 ORFs; 20 tRNAs.	Accession: KR560069
YB07	–	159,862	54.1	*Ackermannviridae*	>98% to *Stenotrophomonas* phage IME-SM1	–	257 ORFs	Accession: MK580972
BUCT555	Hospital sewage; *S. maltophilia* 1207	39,440	61.4	*Podoviridae*	2% coverage to *Myxococcus* phage Mx8	–	57 ORFs	Accession: MW291508
Salva	Soil; *S. maltophilia*	60,789	56.4	*Siphoviridae*	*Stenotrophomonas* phage BUCT548	–	102 ORFs; 1 tRNA.	Accession: MW393850
**Filamentous phages**								
ΦSMA9	Clinical *S. maltophilia* c5	6907	62.4	*Inoviridae*	*Inoviridae* sp. Isolate ctda6	Chronic	Encodes zot-like protein 7 ORFs	[169] NC_007189
ΦSHP1	Environmental *S. maltophilia* P2	6867	61.1	*Inoviridae*	*Stenotrophomonas* phage ΦSMA7	Chronic	Encodes zot-like protein 10 ORFs	[170] NC_010429
ΦSHP2	-	5819	61.5	*Inoviridae*	*Inoviridae* sp. Isolate ctda6	Chronic	Encodes zot-like protein 9 ORFs	[171] NC_015586
ΦSMA6	Environmental *S. maltophilia* Khak84	7648	62.6	*Inoviridae*	Phage ΦSMA9	Chronic	Encodes zot-like protein and putative conjugal transfer protein 11 ORFs	[172] HG315669
ΦSMA7	Environmental *S. maltophilia* Khak84	7069	62.3	*Inoviridae*	*Stenotrophomonas* phage ΦSHP2	Chronic	Encodes zot-like protein 11 ORFs	[172] HG007973
**Phage-derived antimicrobials and PTLBs**								
Lys411 lysozyme	*Xanthomonas oryzae* phage ΦXo411	537	54.2	–	*X. oryzae* phage Xp10 lysozyme	–	No holin required for export to periplasm 124,400 U/mg activity against *S. maltophilia*	[178] DQ408365
Maltocin P28	*S. maltophilia* P28	19,919	66.2	–	–	–	Bactericidal activity against 38 out of 81 strains R-type pyocin structure Mitomycin C inducible, thermolabile, sensitive to proteinase K 23 ORFs	[171] KC787694
Maltocin S16	*S. maltophilia* S16	19,658	66.3	–	–	–	Bactericidal activity against 62 out of 86 strains of *S. maltophilia* Also active against 8 out of 14 *E. coli* strains Mitomycin C inducible, thermolabile, insensitive to proteases Binds LPS as surface receptor 23 ORFs	[180] MH703584

^1^ The top BLASTn hit limited to Viruses (taxid:10239) is recorded. ^a^ Genus was previously classified as *Pseudomonas maltophilia*, which is now known as *Stenotrophomonas maltophilia.* ^b^ Estimated genome size based on PFGE; no sequencing data available. ^c^ Estimated genome size and GC content based on PFGE and HPLC; 16 kb fragment containing morphogenesis genes sequenced. ^d^ Morphology is speculated based on genome characteristics in the absence of electron microscopy.
